# Factors influencing scar formation following Bacille Calmette-Guérin (BCG) vaccination

**DOI:** 10.1016/j.heliyon.2023.e15241

**Published:** 2023-04-06

**Authors:** Paola Villanueva, Nigel W. Crawford, Mariana Garcia Croda, Simone Collopy, Bruno Araújo Jardim, Tyane de Almeida Pinto Jardim, Laurens Manning, Michaela Lucas, Helen Marshall, Cristina Prat-Aymerich, Alice Sawka, Ketaki Sharma, Darren Troeman, Ushma Wadia, Adilia Warris, Nicholas Wood, Nicole L. Messina, Nigel Curtis, Laure F. Pittet

**Affiliations:** a Department of Paediatrics, The University of Melbourne, Parkville, VIC, Australia; b Infection and Immunity, Murdoch Children's Research Institute, Parkville, VIC, Australia; c Infectious Diseases, Royal Children's Hospital Melbourne, Parkville, VIC, Australia; d Department of General Medicine, Royal Children's Hospital Melbourne, Parkville, VIC, Australia; e Immunisation Service, Royal Children's Hospital Melbourne, Parkville, VIC, Australia; f School of Medicine, Federal University of Mato Grosso do Sul, Campo Grande, MS, Brazil; g Department of Pediatrics, Universidade Estadual do Rio de Janeiro, Rio de Janeiro, Brazil; h Carlos Borborema Clinical Research Institute, Fundação de Medicina Tropical Dr. Heitor Vieira Dourado, Manaus, Brazil; i Wesfarmers Centre for Vaccines and Infectious Diseases, Telethon Kids Institute, Perth, WA, Australia; j School of Medicine, University of Western Australia, Perth, WA, Australia; k Department of Infectious Diseases, Fiona Stanley Hospital, Perth, WA, Australia; l Department of Immunology, Sir Charles Gairdner Hospital, Perth, WA, Australia; m Departments of Immunology, Perth Children's Hospital, Perth, WA, Australia; n Department of Immunology, Pathwest, QE2 Medical Centre, Perth, WA, Australia; o Robinson Research Institute and Adelaide Medical School, The University of Adelaide and Department of Paediatrics, The Women's and Children's Health Network, Australia; p Julius Center for Health Sciences and Primary Care, University Medical Center Utrecht, Utrecht University, Utrecht, Netherlands; q Institut d'Investigació Germans Trias i Pujol, Departament de Genètica i Microbiologia, CIBER de enfermedades respiratorias (CIBERES), Instituto de Salud Carlos III, Universitat Autònoma de Barcelona, Catalunya, Badalona, Spain; r Department of Thoracic Medicine, Royal Adelaide Hospital, Adelaide, SA, Australia; s National Centre for Immunisation Research and Surveillance, Westmead, NSW, Australia; t The Children's Hospital at Westmead, Westmead, NSW, Australia; u Medical Research Council Centre for Medical Mycology, University of Exeter, UK; v Great Ormond Street Hospital, London, UK; w The Children's Hospital at Westmead Clinical School, Faculty of Medicine and Health, University of Sydney, NSW, Australia; x Infectious Diseases Unit, Department of Paediatrics, Gynaecology and Obstetrics, Faculty of Medicine, University of Geneva and University Hospitals of Geneva, Geneva, Switzerland

**Keywords:** BCG vaccine, BCG scar, Vaccination technique, Vaccine safety

## Abstract

The prevalence of scar formation following Bacille Calmette-Guérin (BCG) vaccination varies globally. The beneficial off-target effects of BCG are proposed to be stronger amongst children who develop a BCG scar. Within an international randomised trial (‘BCG vaccination to reduce the impact of coronavirus disease 2019 (COVID-19) in healthcare workers’; BRACE Trial), this nested prospective cohort study assessed the prevalence of and factors influencing scar formation, as well as participant perception of BCG scarring 12 months following vaccination . Amongst 3071 BCG-recipients, 2341 (76%) developed a BCG scar. Scar prevalence was lowest in Spain and highest in UK. Absence of post-injection wheal (OR 0.4, 95%CI 0.2–0.9), BCG revaccination (OR 1.7, 95%CI 1.3–2.0), female sex (OR 2.0, 95%CI 1.7–2.4), older age (OR 0.4, 95%CI 0.4–0.5) and study country (Brazil OR 1.6, 95%CI 1.3–2.0) influenced BCG scar prevalence. Of the 2341 participants with a BCG scar, 1806 (77%) did not mind having the scar. Participants more likely to not mind were those in Brazil, males and those with a prior BCG vaccination history. The majority (96%) did not regret having the vaccine.

Both vaccination-related (amenable to optimisation) and individual-related factors affected BCG scar prevalence 12 months following BCG vaccination of adults, with implications for maximising the effectiveness of BCG vaccination.

## 1 Introduction

Bacille Calmette-Guérin (BCG) vaccine is widely administered in over 150 countries to protect children against tuberculosis (TB) [1]. A small characteristic scar at the BCG injection site, which develops over several weeks to months, is considered a normal response and commonly used as a surrogate for effective vaccination [2].

The importance of scar formation is highlighted by studies that link the protective ‘off-target’ (also known as ‘non-specific’) clinical effects of BCG vaccination to the development of a scar [3,4]. In observational studies in low-income countries, BCG-vaccinated children who developed a scar had lower all-cause mortality and fewer hospital admissions than those who did not [[5], [6], [7], [8], [9]]. In addition, the presence and size of BCG scar have been shown to correlate with the magnitude of the immune response to BCG vaccination ^[^^10^^]^.

The prevalence of scar following BCG vaccination varies and the mechanisms underlying this are unclear. Suggested explanations include variation in immune response, the influence of BCG strain and administration technique [9,[11], [12], [13], [14], [15], [16]]. The need for revaccination in scar-negative children is debated [2,13,17]. With increasing interest in BCG vaccination and revaccination for broader uses in both children and adults, it is important to understand more about BCG scarring.

Within an international randomised controlled trial of BCG vaccination to reduce the impact of coronavirus disease 2019 (COVID-19) in healthcare workers (the BRACE trial; ClinicalTrials.gov NCT04327206), this nested prospective cohort study aimed to determine (a) the prevalence of BCG scarring at 12 months following vaccination, (b) the factors influencing scar formation, and (c) participant perception of scarring.

## 2 Methods

### 2.1 Setting and participants

The BRACE trial recruited healthcare workers (HCW) in Australia, Brazil, Spain, the Netherlands and the United Kingdom (UK) from March 2020 to April 2021, and randomised participants to receive BCG vaccine or no BCG. HCW were eligible if working in healthcare settings or having face-to-face contact with patients during the COVID-19 pandemic. Exclusion criteria comprised any contra-indication to BCG, including immunosuppression or previous significant local BCG adverse reaction. Prior BCG vaccination, or previous history of positive tuberculin skin test (TST), were not exclusion criteria. The trial protocol is described in detail elsewhere [18].

### 2.2 Intervention

Participants randomised to BCG received a single dose of BCG-Denmark (AJ Vaccines, Copenhagen), 0.1 ml (corresponding to 2–8 × 10^5^ colony-forming units of *Mycobacterium bovis*, Danish strain 1331) intradermally in the upper arm, using a short (10 mm) bevel needle (25 G to 30 G). If an individual had prior BCG scar evidence at recruitment, the vaccinators were instructed to administer the vaccine or placebo a minimum of 2.5 cm from the original BCG scar. All vaccinators were trained in intradermal delivery of BCG vaccine (Supplemental Material 1). If a post-injection wheal (minimum 7 mm diameter) [19] did not occur immediately, a participant could receive a second vaccine dose. Participants were informed about the normal expected injection site reaction, including likely scar formation. Participants recruited in Australia from March to May 2020 were also required to receive a single intramuscular dose (0.5 ml, pre-filled syringe) of a quadrivalent inactivated influenza vaccine in the contralateral upper arm on the day of randomisation.

### 2.3 Data collection

Data were collected using Research Electronic Data Capture (REDCap) web application [20] including details on demographics, co-morbidities, previous BCG vaccination, previous TST and previous known latent tuberculosis infection (LTBI). Data on the presence of post-injection wheal formation (including injection site photograph), BCG batch, and number of participants BCG vaccinated per vaccinator, were collected. Information on prior BCG vaccination experience (before the BRACE trial) was collected from vaccinators in Brazil and Spain.

**Figure 1:**
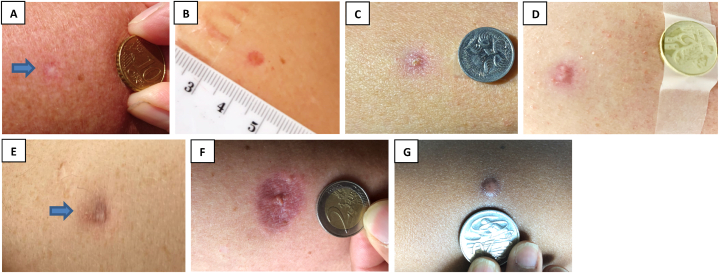
BCG scar description at 12 months. Representative photographs for BCG scar descriptions. *(A) skin colour mark without redness (normal scar formation), (B) red mark, (C) red mark with crusting, (D) red mark with discharge, (E) purple mark, (F) inflamed appearance with surrounding swelling and/or redness, (G) abnormal thick scar.*

Information on injection site scar formation (presence and scar descriptors), vaccine site photographs (with ruler or standard coin for scale; Fig. 1(A-G)) and participant scar perception were solicited from participants using a standardised web-based questionnaire 12 months following vaccination (Supplemental Material 2). Participants who reported an ‘abnormal thick scar’ had injection site photos reviewed by a medical doctor, to assess for keloid scar formation.

### 2.4 Case definitions

The following definitions were used. BCG-revaccination: BCG vaccination in a participant who had any prior BCG vaccination history. Post-injection wheal: a skin wheal of minimum 7 mm diameter immediately following intradermal vaccination [19]. Imperfect BCG vaccine administration: the absence of a post-injection site wheal. Keloid scar: a thick raised scar extending upwards and outwards well beyond the site of vaccination.

### 2.5 Statistical analysis

StataIC 14.0 (Statacorp LP, College Station, TX, USA) was used. BCG scar prevalence at 12 months following BCG vaccination was calculated among participants who provided injection site data. BCG scar prevalence was evaluated by study country and by individual vaccinator (defined as proportion of vaccinees with scar presence at 12 months). To identify factors (participant-related and vaccination-related) associated with BCG scar formation, odds ratio (OR) and 95% confidence intervals (CI) were determined using univariate logistic regression. Post-injection wheal presence, as a potential associated factor, was analysed amongst participants who received one BCG dose only. Significant factors (p-value <0.2) resulting from the univariate logistic regression analysis were included as possible covariates in a multivariate logistic regression model. The model was created using backward stepwise exclusion of factors with p-value >0.05, using sequential model testing. Participant BCG scar descriptors and scar perception were analysed amongst participants with a BCG scar at 12 months following BCG vaccination.

Ethical approval was obtained from The Royal Children's Hospital Human Research Ethics Committee (HREC 62586) with subsequent approvals from all participating sites. All participants provided signed informed consent prior to enrolment.

## 3 Results

**Figure 2:**
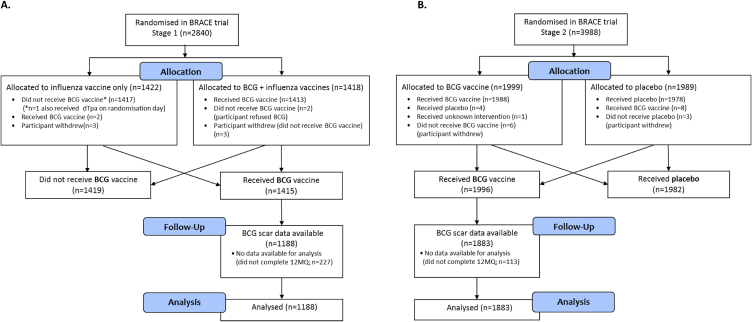
BRACE participants who received BCG in A) Stage 1 and B) Stage 2. Abbreviations: BCG, Bacille Calmette-Guérin; BRACE trial, BCG vaccination to reduce the impact of coronavirus disease 2019 (COVID-19) in healthcare workers; dTpa, diphtheria-tetanus-acellular pertussis vaccine, reduced antigen formulation; 12MQ, 12-month questionnaire.

Of the 3411 participants who were BCG-vaccinated in the BRACE trial, 3071 (90%) provided injection site information in their 12-month questionnaire (Fig. 2 (A and B)). They ranged in age from 18 to 78 years (median 41) and the majority (76%) were female (Table 1).

**Table 1:** Demographics and factors investigated for association with BCG scar formation.

**Factor**	Total BCG		**Scar prevalence**	
			Univariate	Multivariate
	(N=3071)	n/N (%)	OR (95% CI)	OR (95% CI)
Sex				
Male	822	556 (67.6)	1 (reference)	1 (reference)
Female	2249	1785 (79.4)	1.84 (1.53–2.20), p < 0.001	2.00 (1.65–2.42), p < 0.001
Age				
18–49	2144	1733 (80.8)	1 (reference)	1 (reference)
≥50	927	608 (65.6)	0.45 (0.38–0.54), p < 0.001	0.43 (0.35–0.51), p < 0.001
Nutritional status (BMI)				
Normal weight (18.5–24.9)	1266	964 (76.1)	1 (reference)	–
Underweight (<18.5)	35	28 (80.0)	1.25 (0.54–2.90), p = 0.6	
Pre-obesity (25.0–29.9)	1090	820 (75.2)	0.95 (0.79–1.15), p = 0.6	
Obesity class I (30.0–34.9)	419	328 (78.2)	1.13 (0.87–1.47), p = 0.4	
Obesity class II (35.0–39.9)	155	121 (78.1)	1.11 (0.75–1.67), p = 0.6	
Obesity class III (≥40)	58	45 (77.6)	1.08 (0.58–2.04), p = 0.4	
Unknown	48	35 (72.9)	NA	
Smoker				
No	2808	2131 (75.9)	1 (reference)	–
Yes	263	210 (79.8)	1.26 (0.92–1.72), p = 0.2	
Diabetes				
No	2991	2278 (76.2)	1 (reference)	–
Yes	80	63 (78.8)	1.16 (0.67–1.99), p = 0.6	
Chronic respiratory disease				
No	2866	2177 (76.0)	1 (reference)	–
Yes	205	164 (80.0)	1.27 (0.89–1.80), p = 0.2	
Chronic cardiovascular disease				
No	2740	2107 (76.9)	1 (reference)	–
Yes	331	234 (70.7)	0.72 (0.56–0.93), p = 0.01	
Study country				
Australia	1380	1003 (72.7)	0.70 (0.59–0.83), p < 0.001	0.84 (0.70–1.01), p = 0.07
Brazil	1222	1032 (84.4)	2.24 (1.86–2.69), p < 0.001	1.61 (1.29–2.01), p < 0.001
Netherlands	280	187 (66.8)	0.59 (0.46–0.77), p < 0.001	1.01 (0.75–1.36), p = 0.9
Spain	110	52 (47.3)	0.26 (0.18–0.39), p < 0.001	0.31 (0.20–0.46), p < 0.001
UK	79	67 (84.8)	1.76 (0.95–3.28), p = 0.07	1.84 (0.97–3.50), p = 0.06
BCG history				
1st BCG	990	677 (68.4)	1 (reference)	1 (reference)
BCG revaccination	2081	1664 (80.0)	1.85 (1.55–2.19), p < 0.001	1.65 (1.33–2.04), p < 0.001
Previous known LTBI				
No	3031	2309 (76.2)	1 (reference)	–
Yes	23	16 (69.6)	0.71 (0.29–1.74), p = 0.5	
Unknown	17	16 (94.1)	NA	
Previous TST				
Negative/None	2568	1961 (76.4)	1 (reference)	–
Positive (>5 mm)	186	149 (80.1)	1.25 (0.86–1.81), p = 0.2	
Unknown	317	231 (72.9)	NA	
BCG batch				
118006D	591	431 (72.9)	0.80 (0.66–0.99), p = 0.04	–
118017F	820	587 (71.6)	0.71 (0.60–0.86), p < 0.001	
118019D	658	536 (81.5)	1.48 (1.19–1.84), p = 0.001	
119039B	82	70 (85.4)	1.84 (0.99–3.42), p = 0.06	
119053A	631	527 (83.5)	1.75 (1.39–2.20), p < 0.001	
200731–014	245	160 (65.3)	0.56 (0.42–0.73), p < 0.001	
200904–017	35	27 (77.1)	1.05 (0.48–2.33), p = 0.9	
Unknown	9	3 (33.3)	NA	
Co-administered influenza vaccine†				
No	1883	1490 (79.1)	1 (reference)	–
Yes	1188	851 (71.6)	0.67 (0.57–0.79), p < 0.001	
Post-injection wheal*				
Yes	2898	2223 (76.7)	1 (reference)	1 (reference)
No	32	19 (59.4)	0.44 (0.22–0.90), p = 0.03	0.44 (0.21–0.93), p = 0.03
Unknown	141	99 (70.2)	NA	NA
Vaccinator experience				
≥20 vaccinees	2608	2007 (77.0)	1 (reference)	–
0-19 vaccinees	463	334 (72.1)	0.76 (0.61–0.95), p = 0.02	

Abbreviations: BCG, Bacille Calmette-Guérin; BMI, body mass index; OR, odds ratio; LTBI, latent tuberculosis infection; NA, not applicable; TST, tuberculin skin test. *Wheal response (yes/no) analysed for participants who received one BCG dose only. †Stage 1 participants (Australia) were required to receive influenza vaccination on day of randomisation. Significant factors (p-value <0.2) resulting from the univariate logistic regression analysis were included as possible covariates in a multivariate logistic regression model. The model presented in the table was created using backward stepwise exclusion of factors with p-value >0.05, using sequential model testing.

### 3.1 BCG scar prevalence

**Figure 3:**
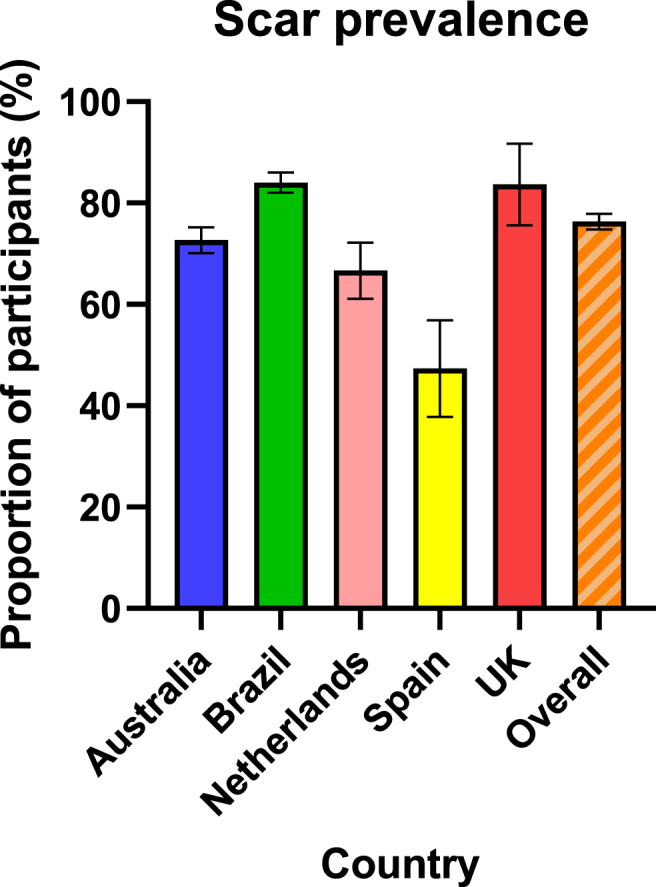
BCG scar prevalence at 12 months by recruitment country, with 95% confidence interval.

Overall, BCG scar prevalence at 12 months was 76% (2341/3071); lowest (47%, 52/110) in Spain and highest (85%, 67/79) in the UK (Fig. 3). Most scars were visible to participants (as opposed to only palpable) (96%, 2254/2341; Table 2) and were reported as a ‘skin colour mark without redness’ by 71% (1651/2341) of participants (Table 3; Fig. 1(A)). Scars were reported as ‘abnormally thick’ by 3% (60/2341). Of these 60 participants, 22 supplied an injection site photo that showed hypertrophic scarring. Two of these (one each in Australia and Brazil) had a keloid scar at their BCG injection site, one of whom had prior predisposition to keloid scarring.

**Table 2:** BCG scar visibility and palpability at 12 months, as reported by participants.

Scar visibility and palpability	n = 2341
Visible only, not palpable	1267 (54%)
Visible and palpable	970 (41%)
Palpable only, not visible	74 (3%)
Visible and palpable with crust	17 (<1%)
Unknown	13 (<1%)

**Table 3:** BCG scar description at 12 months

Scar description	n = 2341
Skin colour mark without redness (normal scar formation)	1651 (71%)
Red mark	602 (26%)
Red mark with crusting	14 (<1%)
Red mark with discharge	3 (<1%)
Purple mark	7 (<1%)
Inflamed appearance with surrounding swelling and/or redness	4 (<1%)
Abnormal thick scar	60 (3%)
Ulcer	0 (0%)

	Total		Scar prevalence	
Factor	BCG		Univariate	Multivariate
	n = 1380	n/N (%)	OR (95% CI)	OR (95% CI)
Sex				
Male	353	219 (62.0)	1 (reference)	1 (reference)
Female	1027	784 (76.3)	1.97 (1.52–2.56), p < 0.001	2.04 (1.54–2.71), p < 0.001
Age				
18–49	912	701 (76.9)	1 (reference)	1 (reference)
≥50	468	302 (64.5)	0.55 (0.43–0.70), p < 0.001	0.41 (0.31–0.54), p < 0.001
Nutritional status (BMI)				
Normal weight (18.5–24.9)	621	471 (75.8)	1 (reference)	–
Underweight (<18.5)	11	7 (63.6)	0.56 (0.16–1.93), p = 0.4	
Pre-obesity (25.0–29.9)	451	311 (69.0)	0.71 (0.54–0.93), p = 0.01	
Obesity class I (30.0–34.9)	163	114 (69.9)	0.74 (0.51–1.09), p = 0.1	
Obesity class II (35.0–39.9)	62	47 (75.8)	1.00 (0.54–1.84), p = 0.9	
Obesity class III (>40)	32	26 (81.3)	1.38 (0.56–3.42), p = 0.5	
Unknown	40	27 (67.5)	NA	
Smoker				
No	1298	948 (73.0)	1 (reference)	–
Yes	82	55 (67.1)	0.75 (0.47–1.21), p = 0.2	
Diabetes				
No	1359	988 (72.7)	1 (reference)	–
Yes	21	15 (71.4)	0.94 (0.36–2.44), p = 0.9	
Chronic respiratory disease				
No	1276	920 (72.1)	1 (reference)	–
Yes	104	83 (79.8)	1.53 (0.93–2.51), p = 0.1	
Chronic cardiovascular disease				
No	1274	934 (73.3)	1 (reference)	–
Yes	106	69 (65.1)	0.68 (0.45–1.03), p = 0.07	
BCG history				
1st BCG	651	463 (71.1)	1 (reference)	1 (reference)
BCG revaccination	729	540 (74.1)	1.16 (0.92–1.47), p = 0.2	1.58 (1.19–2.08), p = 0.001
Previous known LTBI				
No	1353	983 (72.7)	1 (reference)	–
Yes	15	9 (60.0)	0.56 (0.20–1.60), p = 0.3	
Unknown	12	11 (91.7)	NA	
Previous TST				
Negative/None	1021	739 (72.4)	1 (reference)	–
Positive (>5 mm)	115	88 (76.5)	1.24 (0.79–1.96), p = 0.3	
Unknown	244	176 (72.1)	NA	
BCG batch				
118006D	591	431 (72.9)	1.02 (0.80–1.30), p = 0.9	–
118017F	789	572 (72.5)	0.98 (0.77–1.24), p = 0.9	
Co-administered influenza vaccine†				
No	192	152 (79.2)	1 (reference)	1 (reference)
Yes	1188	851 (71.6)	0.66 (0.46–0.96), p = 0.03	0.57 (0.39–0.84), p < 0.01
Post-injection wheal*				
Yes	1231	899 (73.0)	1 (reference)	1 (reference)
No	19	11 (57.9)	0.51 (0.20–1.27), p = 0.1	0.34 (0.13–0.90), p = 0.03
Unknown	130	93 (71.5)	NA	–
Vaccinator experience				
≥20 vaccinees	1199	864 (72.1)	1 (reference)	–
0-19 vaccinees	181	139 (76.8)	1.28 (0.89–1.85), p = 0.2	

	Total		Scar prevalence	
Factor	BCG		Univariate	Multivariate
	n = 1222	n/N (%)	OR (95% CI)	OR (95% CI)
Sex				
Male	350	279 (79.7)	1 (reference)	1 (reference)
Female	872	753 (86.3)	1.61 (1.16–2.23), p < 0.01	1.65 (1.18–2.30), p < 0.01
Age				
18–49	977	856 (87.6)	1 (reference)	1 (reference)
≥50	245	176 (71.8)	0.36 (0.26–0.51), p < 0.001	0.37 (0.26–0.52), p < 0.001
Nutritional status (BMI)				
Normal weight (18.5–24.9)	387	327 (84.4)	1 (reference)	–
Underweight (<18.5)	18	15 (83.3)	0.59 (0.26–3.27), p = 0.9	
Pre-obesity (25.0–29.9)	498	418 (83.9)	0.96 (0.67–1.38), p = 0.8	
Obesity class I (30.0–34.9)	212	187 (88.2)	1.37 (0.83–2.26), p = 0.2	
Obesity class II (35.0–39.9)	81	66 (81.5)	0.81 (0.43–1.51), p = 0.5	
Obesity class III (>40)	21	14 (66.7)	0.37 (0.14–0.95), p = 0.04	
Unknown	5	5 (100.0)	NA	
Smoker				
No	1090	916 (84.0)	1 (reference)	–
Yes	132	116 (87.9)	1.38 (0.80–2.38), p = 0.3	
Diabetes				
No	1167	988 (84.7)	1 (reference)	–
Yes	55	44 (80.0)	0.72 (0.37–1.43), p = 0.4	
Chronic respiratory disease				
No	1162	980 (84.3)	1 (reference)	–
Yes	60	52 (86.7)	1.21 (0.56–2.58), p = 0.6	
Chronic cardiovascular disease				
No	1047	894 (85.4)	1 (reference)	–
Yes	175	138 (78.9)	0.64 (0.43–0.95), p = 0.03	
BCG history				
1st BCG	40	31 (77.5)	1 (reference)	–
BCG revaccination	1182	1001 (84.7)	1.61 (0.75–3.43), p = 0.2	
Previous known LTBI				
No	1218	1028 (84.4)	1 (reference)	–
Yes	1	1 (100.0)	NA†	
Unknown	3	3 (100.0)	NA	
Previous TST				
Negative/None	1150	970 (84.3)	1 (reference)	–
Positive (>5 mm)	42	38 (90.5)	1.76 (0.62–5.00), p = 0.3	
Unknown	30	24 (80.0)	NA	
BCG batch				
118019D	658	536 (81.5)	0.60 (0.44–0.83), p < 0.01	0.63 (0.45–0.87), p < 0.01
119039B	3	3 (100.0)	NA†	NA†
119053 A	557	491 (88.2)	1.71 (1.23–2.35), p = 0.001	1.58 (1.13–2.19), p < 0.01
Unknown	4	2 (50.0)	NA	NA
Post-injection wheal*				
Yes	1212	1024 (84.4)	1 (reference)	
No	6	6 (100.0)	NA†	
Unknown	4	2 (50.0)	NA	
Vaccinator experience				
≥20 vaccinees	1098	926 (84.3)	1 (reference)	–
0–19 vaccinees	124	106 (85.5)	1.09 (0.65–1.85), p = 0.7	

#### 3.1.1 Scar prevalence according to vaccinator

**Figure 4:**
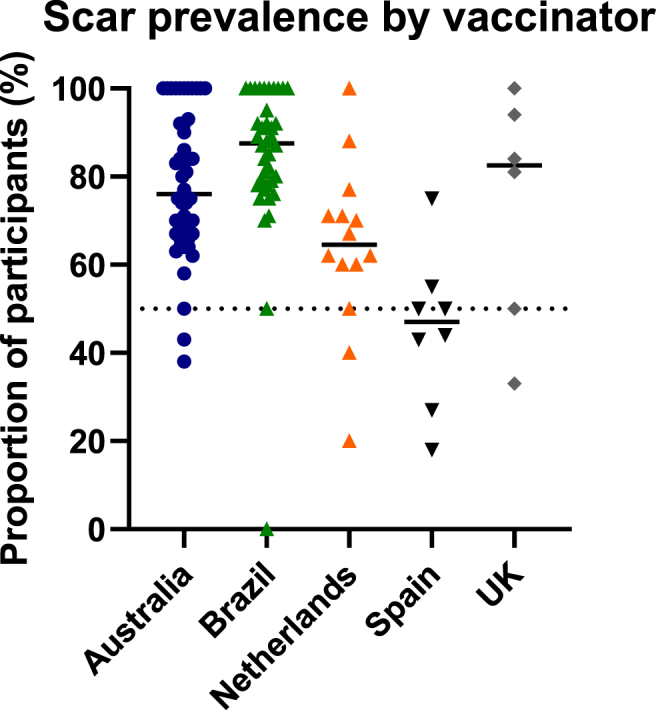
Scar prevalence by vaccinator, grouped by study country. Proportion of scar positive vaccinees at 12 months, per vaccinator. Each data point represents an individual vaccinator. Horizontal lines represent medians.

Amongst the total of 114 vaccinators, those whose vaccinees had a scar prevalence greater than 50% at 12 months, were more prevalent in Brazil (36/38, 95% of vaccinators) and least prevalent in Spain (2/8, 25% of vaccinators) (Fig. 4). In Brazil, 7/38 (18%) vaccinators had prior experience of working in BCG clinics, in addition to specific vaccination training for the BRACE trial. They vaccinated 33% of participants in Brazil. In Spain, none of the 8 vaccinators reported prior experience of working in BCG clinics.

### 3.2 Factors associated with the development of BCG scar

In the univariate analysis, BCG scar formation at 12 months was more common amongst female participants, those with a history of prior BCG vaccination, participants in Brazil, and those vaccinated with certain BCG batches (Table 1). BCG scar formation was less likely in older participants, those with chronic cardiovascular disease, those who had influenza vaccine co-administered, those with imperfect BCG vaccine administration (absence of post-injection wheal), those vaccinated by vaccinators who administered fewer BCG vaccines in the trial, and those vaccinated in Australia, Netherlands or Spain.

In the multivariate analysis, older age group (OR 0.43, 95% CI 0.35–0.51), female sex (OR 2.00, 95%CI 1.65–2.42), study country (Brazil OR 1.61, 95% CI 1.29–2.01; Spain OR 0.31, 95% 0.20–0.46), BCG revaccination (OR 1.65, 95% CI 1.33–2.04), and absence of post-injection wheal (OR 0.44, 95% CI 0.21–0.93) influenced BCG scar prevalence (Table 1).

There was no significant association in either analysis with participant nutritional status, smoking, certain co-morbidities, previous positive TST or LTBI (Table 1).

Sensitivity analyses using participants only in Australia (supplemental Table S1) and those only in Brazil (supplemental Table S2) (the countries with the largest number of participants) also showed, in multivariate analyses, that sex, age group and BCG revaccination influenced BCG scar prevalence.

### 3.3 Participant scar perception

**Figure 5:**
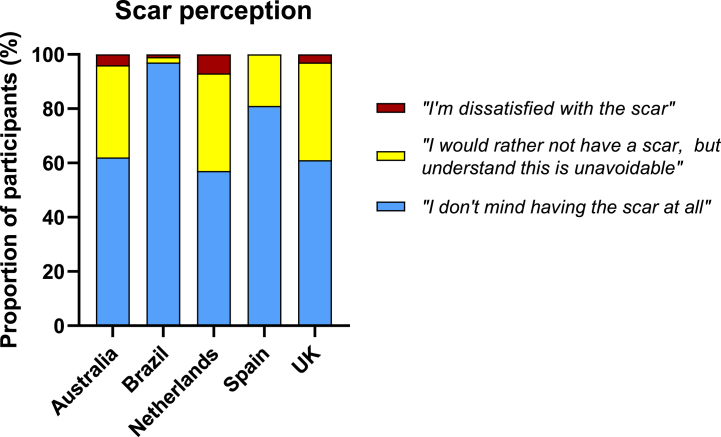
BCG scar perception at 12 months by study country.

Amongst participants with a BCG scar at 12 months, 1806 (77%) ‘[did] not mind having the scar at all’, 472 (20%) would ‘rather not have a scar but [understood] this [was] unavoidable’, and 59 (3%) were ‘dissatisfied with the scar’ (Supplemental Material 3). Brazil had the highest proportion of participants (97%) who ‘[did] not mind having the scar at all’ (Fig. 5). Those BCG revaccinated were more likely to accept scarring, compared with those receiving it for the first time (1382/1661 (83%) vs 424/676 (63%), p < 0.001). Males were more likely to ‘not mind having the scar at all’, compared with females (475/553 (89%) vs 1331/1784 (75%), p < 0.001).

Of the 59 participants who were ‘dissatisfied with the scar’, 46 (78%) reported this was because the scar was ‘worse than [I] expected’, 10 (17%) ‘did not expect to have a scar’, and 3 (5%) reported other reasons. Other reasons were related to scar location, odd and different appearance (purple/red scar colour) to that expected. The majority of participants with a scar at 12 months (96%, 2242/2341) ‘[did] not regret having the vaccine [because of the scar]’.

## 4 Discussion

In this large international study, we found that both vaccination-related and individual-related factors influence scar formation following BCG vaccination.

Studies of scar prevalence following BCG vaccination of adults are scarce and limited by small numbers of vaccinees. In our study of more than 3000 vaccinated healthcare workers, 76% of BCG-Denmark-vaccinated participants reported the presence of a scar at 12 months, with significant differences by country. This is lower than the scar prevalence of 99% reported amongst 175 healthcare students who were BCG-vaccinated 12 months prior with BCG-Denmark in Sweden [21]. Notably, the students were younger (mean age 24 years old) compared with those in our study (mean 42 years old). In children, reported scar prevalence varies from 99% [11] (BCG-Denmark) to as low as 52% [8] (BCG-Russia). Studies have implicated mainly vaccine-related factors such as BCG strain, vaccination route and dose [[10], [11],^15^,[22], [23], [24], [25], [26], [27]].

BCG strains, derived from the original *M. bovis* BCG strain first used in 1921, have acquired phenotypic and genotypic variations during decades of *in vitro* culturing under diverse conditions in laboratories around the world [28,29]. Studies have shown different immunological responses and different mycobacterial viability according to strain [[30], [31], [32], [33]].

We found that age, sex, study country, prior history of BCG vaccination and presence of post-injection wheal may all influence the development of a BCG scar. Vaccination technique and vaccinator experience have previously been reported to affect the local BCG injection site reaction in children [9,11,12,15,25]. Intradermal BCG vaccination is a difficult technique to master and the presence of a post-injection wheal is a marker of intradermal delivery [19]. Consistent with studies in children, we found the absence of a wheal was associated with a decreased likelihood of scar formation, highlighting the importance of a correct vaccine administration. We found an association between number of vaccines given by vaccinators and the likelihood of BCG scar formation, although this was no longer significant in the multivariate analysis. However, different levels of background experience existed amongst vaccinators, in addition to the training received specifically for the trial. For example, in Brazil, the only study country with an ongoing universal neonatal BCG immunisation program, a third of trial participants were vaccinated by vaccinators who had additional prior experience of working in BCG clinics. Consistent with this hypothesis, more vaccinators in Brazil than any other study country had a scar prevalence of >50% amongst their participants, whereas Spain (with the lowest BCG scar prevalence) had the least proportion of such vaccinators.

The higher likelihood of BCG scarring in participants with prior BCG vaccination history may relate to an underlying immunological boosting phenomenon, as BCG revaccination has been associated with more pronounced local injection site reactions [34], larger scar size [35] and enhanced protective effects (against respiratory tract infections) in studies in adolescents [36] and adults [37]. BCG injection site reactions (presence and size) have been shown to correlate with the magnitude of the mycobacteria-specific T-cell immune responses [10] as well as specific and heterologous cytokine responses [9] *in vitro*.

The decreased likelihood of BCG scarring amongst older participants and males may also relate to differing immune responses according to age and sex [38,39]. For older participants, immunosenescence may play a role [40]. Our finding accords with another study comparing individuals BCG-vaccinated at greater than 60 years old with younger adults in Malawi [35].

Sex-differential BCG scar prevalence has been described in two studies in children, showing lower scar formation amongst girls compared with boys [8,41], but not in four other studies [15,21,25,35]. Sex-related differences in beneficial effects of BCG vaccination have also been reported amongst infants; some studies showing the beneficial effects favour the male sex (randomised controlled trials in Uganda [42] and Guinea-Bissau [43]) and others the female sex [6,7,44], Nonetheless, in interpreting our finding of decreased likelihood of scarring amongst males compared with females, we acknowledge the possibility of gender-related differences in reporting behaviour in our study, as men have been shown to underreport on health-related matters and thus may be less likely to notice or report a small scar on their arm [45]. Moreover, the majority of participants in our study were female.

Scar perception differed by country, prior BCG scar experience and sex. Brazil, a high TB prevalence country, had the highest proportion of participants who accepted scarring. This may be due to the active national infant BCG vaccination program normalising BCG scars in the population. There may also be differences in cosmetic scar appearance according to ethnic skin type [46,47]. Males were also less concerned with their BCG scar, consistent with a smaller study assessing scar acceptance amongst high-school-aged children in the UK [41].

This study has some limitations. Scar size was not assessed, and this has previously been shown to correlate with the extent of the underlying immune response [10] and the beneficial off target (non-specific) effects in infants [9,48]. Scar prevalence at 12 months was assessed by participants, which may affect accuracy of scar detection (the self-reporting nature may explain the observed sex-difference), although they were HCW previously informed of the expected injection site reaction at recruitment and were asked to provide injection site photographs. Furthermore, potential confounding factors could include vaccinators’ prior BCG vaccination experience, potential variances in vaccine administration technique, as well as BCG batch.

The strengths of this study include the prospective data collection of vaccine site reactions in a large number of individuals across multiple countries, vaccinated with the same BCG strain.

Our findings have implications for BCG vaccination campaigns as well as the growing number of trials into the beneficial off-target effects of BCG in both adults and children [[49], [50], [51], [52], [53]]. Optimising vaccine-related factors, particularly correct intradermal administration leading to a wheal, can increase the likelihood of scar development and consequent protective effects of BCG vaccination.

## 5 Conclusion

BCG scar prevalence following BCG vaccination in adults was affected by several vaccination-related (vaccine technique, prior BCG, study site) and individual-related (sex, age at vaccination) factors. Although participant BCG scar perception varied by country, sex and prior BCG vaccination, the vast majority of participants did not regret having the vaccine.

## Author contribution statement

Conceptualisation or design of the work: Paola Villanueva, Laure F. Pittet, Nicole L. Messina and Nigel Curtis. Acquisition of data: all authors. Analysis or interpretation of data: Paola Villanueva and Laure F. Pittet. Original drafting: Paola Villanueva. Revising, editing, and final approval of the manuscript: all authors.

## Data availability statement

Data included in article/supp. material/referenced in article.

## References

[1] Zwerling A., Behr M.A., Verma A., Brewer T.F., Menzies D., Pai M. (2011). The BCG World Atlas: a database of global BCG vaccination policies and practices. PLoS Med..

[2] Jason J., Archibald L.K., Nwanyanwu O.C., Kazembe P.N., Chatt J.A., Norton E., Dobbie H., Jarvis W.R. (2002). Clinical and immune impact of Mycobacterium bovis BCG vaccination scarring. Infect. Immun..

[3] Roth A., Gustafson P., Nhaga A., Djana Q., Poulsen A., Garly M.-L., Jensen H., Sodemann M., Rodriques A., Aaby P. (2005). BCG vaccination scar associated with better childhood survival in Guinea-Bissau. Int. J. Epidemiol..

[4] Timmermann C.A., Biering-Sørensen S., Aaby P., Fisker A.B., Monteiro I., Rodrigues A., Benn C.S., Ravn H. (2015). Tuberculin reaction and BCG scar: association with infant mortality. Trop. Med. Int. Health : TM & IH.

[5] Benn C.S., Roth A., Garly M.L., Fisker A.B., Schaltz-Buchholzer F., Timmermann A., Berendsen M., Aaby P. (2020). BCG scarring and improved child survival: a combined analysis of studies of BCG scarring. J. Intern. Med..

[6] Garly M.L., Martins C.L., Balé C., Baldé M.A., Hedegaard K.L., Gustafson P., Lisse I.M., Whittle H.C., Aaby P. (2003). BCG scar and positive tuberculin reaction associated with reduced child mortality in West Africa. A non-specific beneficial effect of BCG?. Vaccine.

[7] Roth A., Sodemann M., Jensen H., Poulsen A., Gustafson P., Weise C., Gomes J., Djana Q., Jakobsen M., Garly M.L., Rodrigues A., Aaby P. (2006). Tuberculin reaction, BCG scar, and lower female mortality. Epidemiology.

[8] Storgaard L., Rodrigues A., Martins C., Nielsen B.U., Ravn H., Benn C.S., Aaby P., Fisker A.B. (2015). Development of BCG scar and subsequent morbidity and mortality in rural Guinea-bissau. Clin. Infect. Dis..

[9] Schaltz-Buchholzer F., Berendsen M., Roth A., Jensen K.J., Bjerregaard-Andersen M., Kjær Sørensen M., Monteiro I., Aaby P., Stabell Benn C. (2020). BCG skin reactions by 2 months of age are associated with better survival in infancy: a prospective observational study from Guinea-Bissau. BMJ Glob. Health.

[10] Pittet L.F., Fritschi N., Tebruegge M., Dutta B., Donath S., Messina N.L., Casalaz D., Hanekom W.A., Britton W.J., Robins-Browne R., Curtis N., Ritz N. (2022). Bacillus calmette-Guérin skin reaction predicts enhanced mycobacteria-specific T-cell responses in infants: a post Hoc analysis of a randomized controlled trial. Am. J. Respir. Crit. Care Med..

[11] Funch K.M., Thysen S.M., Rodrigues A., Martins C.L., Aaby P., Benn C.S., Fisker A.B. (2018). Determinants of BCG scarification among children in rural Guinea-Bissau: a prospective cohort study. Hum. Vaccines Immunother..

[12] Birk N.M., Nissen T.N., Ladekarl M., Zingmark V., Kjærgaard J., Jensen T.M., Jensen S.K., Thøstesen L.M., Kofoed P.E., Stensballe L.G., Andersen A., Pryds O., Nielsen S.D., Benn C.S., Jeppesen D.L. (2017). The association between Bacillus Calmette-Guérin vaccination (1331 SSI) skin reaction and subsequent scar development in infants. BMC Infect. Dis..

[13] Rani S.H., Vijayalakshmi V., Sunil K., Lakshmi K.A., Suman L.G., Murthy K.J. (1998). Cell mediated immunity in children with scar-failure following BCG vaccination. Indian Pediatr..

[14] Santiago E.M., Lawson E., Gillenwater K., Kalangi S., Lescano A.G., Du Quella G., Cummings K., Cabrera L., Torres C., Gilman R.H. (2003). A prospective study of bacillus Calmette-Guérin scar formation and tuberculin skin test reactivity in infants in Lima, Peru. Pediatrics.

[15] Jensen T.M., Jensen S.K., Birk N.M., Rieckmann A., Hoffmann T., Benn C.S., Jeppesen D.L., Pryds O., Nissen T.N. (2021). Determinants of bacille calmette-Guérin scarification in Danish children. Heliyon.

[16] Shabariah R., Hatta M., Idris I., Santoso A., Patellongi I., Permatasari T.A.E., Farsida, Islam A.A., Natzir R., Wahyudin B., Warsinggih & Emilda (2021). Comparison TLR2 and TLR4 serum levels in children with pulmonary and extrapulmonary tuberculosis with and without a Bacillus Calmette-Guérin (BCG) scar. Journal of Clinical Tuberculosis and Other Mycobacterial Diseases.

[17] World Health Organization (2018). BCG vaccines: WHO position paper February 2018. Wkly. Epidemiol. Rec..

[18] Pittet L.F., Messina N.L., Gardiner K., Orsini F., Abruzzo V., Bannister S., Bonten M., Campbell J.L., Croda J., Dalcolmo M., Elia S., Germano S., Goodall C., Gwee A., Jamieson T., Jardim B., Kollmann T.R., Guimarães Lacerda M.V., Lee K.J., Legge D., Lucas M., Lynn D.J., McDonald E., Manning L., Munns C.F., Perrett K.P., Prat Aymerich C., Richmond P., Shann F., Sudbury E., Villanueva P., Wood N.J., Lieschke K., Subbarao K., Davidson A., Curtis N. (2021). BCG vaccination to reduce the impact of COVID-19 in healthcare workers: protocol for a randomised controlled trial (BRACE trial). BMJ Open.

[19] Australian Technical Advisory Group on Immunisation (ATAGI) (2018).

[20] Harris P.A., Taylor R., Thielke R., Payne J., Gonzalez N., Conde J.G. (2009). Research electronic data capture (REDCap)—a metadata-driven methodology and workflow process for providing translational research informatics support. J. Biomed. Inf..

[21] Fjällbrant H., Ridell M., Larsson L.O. (2008). BCG scar and tuberculin reactivity in children and adults. Scand. J. Infect. Dis..

[22] Anderson E.J., Webb E.L., Mawa P.A., Kizza M., Lyadda N., Nampijja M., Elliott A.M. (2012). The influence of BCG vaccine strain on mycobacteria-specific and non-specific immune responses in a prospective cohort of infants in Uganda. Vaccine.

[23] Frankel H., Byberg S., Bjerregaard-Andersen M., Martins C.L., Aaby P., Benn C.S., Fisker A.B. (2016). Different effects of BCG strains - a natural experiment evaluating the impact of the Danish and the Russian BCG strains on morbidity and scar formation in Guinea-Bissau. Vaccine.

[24] Palmer C.E., Edwards P.Q. (1953). [Variation in technique of intracutaneous BCG vaccination]. Br. Med. J..

[25] Roth A., Sodemann M., Jensen H., Poulsen A., Gustafson P., Gomes J., Djana Q., Jakobsen M., Garly M.L., Rodrigues A., Aaby P. (2005). Vaccination technique, PPD reaction and BCG scarring in a cohort of children born in Guinea-Bissau 2000-2002. Vaccine.

[26] Valenzuela M.T., Ferrer X., Leal I., Pacheco M., Castillo N., Cumsille F. (1998). [Comparative study of the efficacy of two types of BCG vaccines administered in different doses]. Rev. Med. Chile.

[27] Vallishayee R.S., Shashidhara A.N., Bunch-Christensen K., Guld J. (1974). Tuberculin sensitivity and skin lesions in children after vaccination with 11 different BCG strains. Bull. World Health Organ..

[28] Behr M.A. (2002). BCG--different strains, different vaccines?. Lancet Infect. Dis..

[29] Oettinger T., Jørgensen M., Ladefoged A., Hasløv K., Andersen P. (1999). Development of the Mycobacterium bovis BCG vaccine: review of the historical and biochemical evidence for a genealogical tree. Tuber. Lung Dis..

[30] Ritz N., Dutta B., Donath S., Casalaz D., Connell T.G., Tebruegge M., Robins-Browne R., Hanekom W.A., Britton W.J., Curtis N. (2012). The influence of bacille Calmette-Guerin vaccine strain on the immune response against tuberculosis: a randomized trial. Am. J. Respir. Crit. Care Med..

[31] da Silva A.S.M., Albuquerque L.H.P., de Ponte C.G.G., de Almeida M.R., de Faria S.E.R., Ribeiro M.D.S., Pereira E., Antas P.R.Z. (2022). Time to face the proofs: the BCG Moreau vaccine promotes superior inflammatory cytokine profile in vitro when compared with Russia, Pasteur, and Danish strains. Hum. Vaccines Immunother..

[32] Angelidou A., Conti M.G., Diray-Arce J., Benn C.S., Shann F., Netea M.G., Liu M., Potluri L.P., Sanchez-Schmitz G., Husson R., Ozonoff A., Kampmann B., van Haren S.D., Levy O. (2020). Licensed Bacille Calmette-Guérin (BCG) formulations differ markedly in bacterial viability, RNA content and innate immune activation. Vaccine.

[33] Fine P.E.M., Carneiro I.A.M., Milstien J.B., Clements C.J., World Health O. (1999).

[34] Villanueva P., Wadia U., Crawford N., Messina N.L., Kollmann T.R., Lucas M., Manning L., Ricmond P., Pittet L.F., Curtis N. (2022). Revaccination with Bacille Calmette-Guérin (BCG) is associated with an increased risk of abscess and lymphadenopathy. npj Vaccines.

[35] Floyd S., Ponnighaus J.M., Bliss L., Warndorff D.K., Kasunga A., Mogha P., Fine P.E. (2000). BCG scars in northern Malawi: sensitivity and repeatability of scar reading, and factors affecting scar size. Int. J. Tubercul. Lung Dis..

[36] Nemes E., Geldenhuys H., Rozot V., Rutkowski K.T., Ratangee F., Bilek N., Mabwe S., Makhethe L., Erasmus M., Toefy A., Mulenga H., Hanekom W.A., Self S.G., Bekker L.G., Ryall R., Gurunathan S., DiazGranados C.A., Andersen P., Kromann I., Evans T., Ellis R.D., Landry B., Hokey D.A., Hopkins R., Ginsberg A.M., Scriba T.J., Hatherill M. Prevention of M. (2018). Tuberculosis infection with H4:IC31 vaccine or BCG revaccination. N. Engl. J. Med..

[37] Giamarellos-Bourboulis E.J., Tsilika M., Moorlag S., Antonakos N., Kotsaki A., Domínguez-Andrés J., Kyriazopoulou E., Gkavogianni T., Adami M.E., Damoraki G., Koufargyris P., Karageorgos A., Bolanou A., Koenen H., van Crevel R., Droggiti D.I., Renieris G., Papadopoulos A., Netea M.G. (2020). Activate: randomized clinical trial of BCG vaccination against infection in the elderly. Cell.

[38] Aaby P., Benn C.S., Flanagan K.L., Klein S.L., Kollmann T.R., Lynn D.J., Shann F. (2020). The non-specific and sex-differential effects of vaccines. Nat. Rev. Immunol..

[39] Flanagan K.L., Fink A.L., Plebanski M., Klein S.L. (2017). Sex and gender differences in the outcomes of vaccination over the life course. Annu. Rev. Cell Dev. Biol..

[40] Crooke S.N., Ovsyannikova I.G., Poland G.A., Kennedy R.B. (2019). Immunosenescence and human vaccine immune responses. Immun. Ageing : I & A.

[41] Fang J.W., Ko B.M., Wilson J.A. (1993). BCG vaccination scars: incidence and acceptance amongst British high-school children. Child Care Health Dev..

[42] Prentice S., Nassanga B., Webb E.L., Akello F., Kiwudhu F., Akurut H., Elliott A.M., Arts R.J.W., Netea M.G., Dockrell H.M., Cose S. (2021). BCG-induced non-specific effects on heterologous infectious disease in Ugandan neonates: an investigator-blind randomised controlled trial. Lancet Infect. Dis..

[43] Biering-Sørensen S., Jensen K.J., Monterio I., Ravn H., Aaby P., Benn C.S. (2018). Rapid protective effects of early BCG on neonatal mortality among low birth weight boys: observations from randomized trials. J. Infect. Dis..

[44] Stensballe L.G., Nante E., Jensen I.P., Kofoed P.E., Poulsen A., Jensen H., Newport M., Marchant A., Aaby P. (2005). Acute lower respiratory tract infections and respiratory syncytial virus in infants in Guinea-Bissau: a beneficial effect of BCG vaccination for girls community based case-control study. Vaccine.

[45] Caroli E., Weber-Baghdiguian L. (2016). Self-reported health and gender: the role of social norms. Soc. Sci. Med..

[46] Rawlings A.V. (2006). Ethnic skin types: are there differences in skin structure and function?1. Int. J. Cosmet. Sci..

[47] Bayat A., McGrouther D.A., Ferguson M.W. (2003). Skin scarring. BMJ.

[48] Bohlbro A.S., Mendes A.M., Sifna A., Patsche C.B., Soelberg M.E.S., Gomes V., Wejse C., Rudolf F. (2022). BCG is protective against death in male but not female patients with pulmonary tuberculosis in Guinea-Bissau. Trans. R. Soc. Trop. Med. Hyg..

[49] Angelidou A., Pittet L.F., Faustman D., Curtis N., Levy O. (2022). BCG vaccine's off-target effects on allergic, inflammatory, and autoimmune diseases: worth another shot?. J. Allergy Clin. Immunol..

[50] Kühtreiber W.M., Tran L., Kim T., Dybala M., Nguyen B., Plager S., Huang D., Janes S., Defusco A., Baum D., Zheng H., Faustman D.L. (2018). Long-term reduction in hyperglycemia in advanced type 1 diabetes: the value of induced aerobic glycolysis with BCG vaccinations. npj vaccines.

[51] Ristori G., Faustman D., Matarese G., Romano S., Salvetti M. (2018). Bridging the gap between vaccination with Bacille Calmette-Guérin (BCG) and immunological tolerance: the cases of type 1 diabetes and multiple sclerosis. Curr. Opin. Immunol..

[52] Curtis N., Sparrow A., Ghebreyesus T.A., Netea M.G. (2020). Considering BCG vaccination to reduce the impact of COVID-19. Lancet.

[53] Moorlag S., Arts R.J.W., van Crevel R., Netea M.G. (2019). Non-specific effects of BCG vaccine on viral infections. Clin. Microbiol. Infection.

