# Supplementary material for: Factors influencing scar formation following Bacille Calmette-Guérin (BCG) vaccination
Source: Heliyon. 2023 Apr 6;9(4):e15241. doi: 10.1016/j.heliyon.2023.e15241 (PMC10126857; doi:10.1016/j.heliyon.2023.e15241)
Supplement: Multimedia component 1 [file mmc1.pdf]

Standard Operating Procedures: BRACE – Vaccination Station

**Title:**	BRACE Global SOP Vaccination Station
**Version:**	5.0
**Date:**	26 Sep 2020
**Institution Name**	Murdoch Children's Research Institute
**Authors**	Veronica Abruzzo, Joyce Chan and Casey Goodall
**Resources**	Australian Immunisation Handbook: BCG vaccination procedures, 2018 ATAGI<br>BCG vaccine AJV: Package leaflet information for the user (AJVaccines)

Pre-clinic steps:

1. Ensure privacy for the participant if required (e.g. upper left arm not accessible due to clothing).
2. Ensure blinding facilities (e.g. curtain) are available; as syringes will need to be drawn up in session after randomisation and the participant **MUST NOT** see whether the injection they are receiving contains the BCG vaccine or placebo.
3. Ensure that facilities for the treatment of any potential anaphylactic reaction (extremely rare) are available (i.e. vials of IM adrenaline and appropriate needles) and that all staff know the emergency numbers to call at your site [insert site-specific emergency numbers].
4. Locate batch ID numbers for the following and affix to the tablet or keep nearby for easy reference as you will refer to this several times during data entry:
    a. BCG vaccine and diluent
    b. Placebo
5. A single dose of BCG vaccine SSI or matched placebo will be given to all participants who are randomised. The adult dose is 0.1 mL (of BCG vaccine SSI or 0.9% NaCl) injected intradermally over the distal insertion of the deltoid muscle onto the humerus (approximately one third down the upper arm). There are no allowable dose modifications. See below for storage conditions for the vaccine and placebo.

   The vaccine/placebo **must** be given in the LEFT arm. If a previous BCG scar is visible, the new injection **must** be at least 2.5 cm away from all previous BCG scars.

   NOTE FOR BRAZIL SITES – In Brazil, BCG may be given in either the left or right arm. Therefore, vaccinators in Brazil MUST document in which arm the BCG was given.

6. **Resuspension of BCG with diluent**
   A video demonstration of BCG resuspension is available.

    a. The rubber stopper is wiped with an antiseptic solution, such as 2% chlorhexidine & 70% alcohol, allowing the alcohol to fully evaporate before the stopper is penetrated with the syringe needle.

b. The BCG is re-suspended using the diluent provided according to the product directions then carefully inverted a few times to produce uniform resuspension of the lyophilised BCG.
c. Do not shake the vial.
d. The staff member who re-suspends the BCG will label the vial with the date, time of reconstitution and their initials.
e. To ensure a uniform suspension, and therefore dose, the vial will be gently swirled before drawing up each dose. When drawn up into the syringe, the reconstituted vaccine should appear homogeneous, slightly opaque and colourless.
f. Each vial can be kept for up to 4 hours after resuspension.
g. During this time, the vial is kept at 2°C - 8°C. Each vial is discarded after 4 hours, or when the vial is empty, whichever occurs first.
h. Immunisers are asked to monitor vial reconstitution/opening for own reference.
i. Each vial of BCG contains up to 10 adult doses.
j. **NEVER administer the whole vial.**

7. **Sodium Chloride 0.9% placebo**
    a. During each recruitment session, sodium chloride 0.9% will be decanted using aseptic technique into an empty sterile amber glass vial. This amber glass vial should look the same as the BCG vial. Multiple participants are to be dosed from the same vial (i.e. placebo and BCG are both drawn from multi-dose vial to ensure processes are consistent).
    b. The rubber stopper is wiped with an antiseptic solution, such as 2% chlorhexidine & 70% alcohol, allowing the alcohol to fully evaporate before the stopper is penetrated with the syringe needle.
    c. The investigator who prepares the sodium chloride for injection will record the date, time of the preparation and their initials on the labels provided and affix these labels to the placebo vial.
    d. The prepared sodium chloride for placebo can be kept for up to 24 hours. All prepared syringes or vials unused at the end of a vaccination session will be discarded.

BRACE Data Capture and Vaccine Administration Steps:

8. Open Web Browser and go to https://redcap.mcri.edu.au/.

9. Log onto REDCap database with your user ID and Password.

10. Greet participant at door.

11. Go to "**My projects**", Select "**BRACE EConsent and Randomisation**"

12. Click on "**Record Status Dashboard**". Search for or scroll down to select the participant's "**Record ID**" number.

Alternatively, you can go to ‘**Add / Edit Records**’ (left side of home screen, written in red text) and search for the participant using their name, Record ID, email address, mobile number etc.

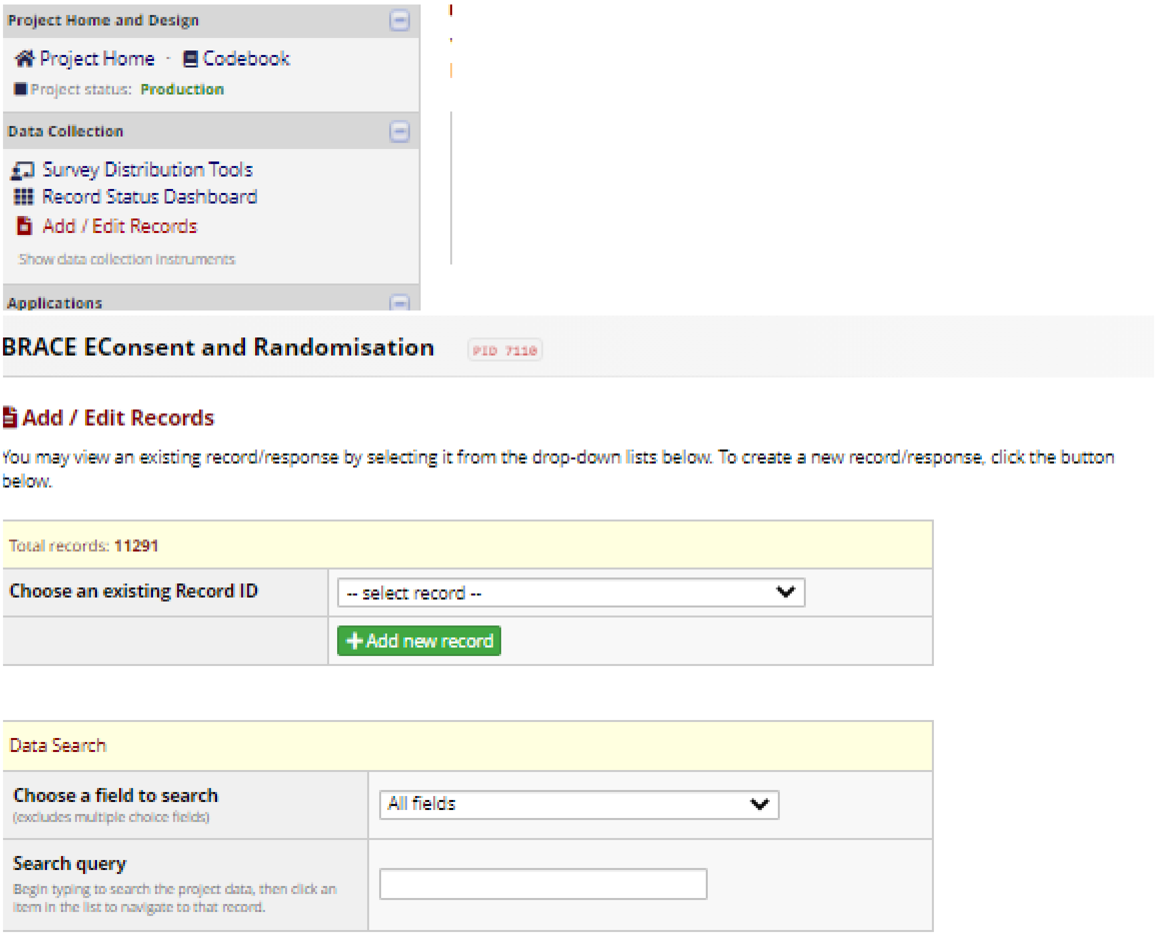

13. Check that the participant’s name and email address are correct in their REDCap record.

    Note – some participants may have duplicate records if they attempted the online pre-consent process more than once. If this is the case, check that you are using the correct record – there should be a green circle next to the Randomisation form.

14. Open the “**Clinic Visit Vaccination**” form. Do NOT allow participant or blinded team members to view this form and ensure you log out of REDCap before allowing other team members to use the same iPad/tablet/computer.

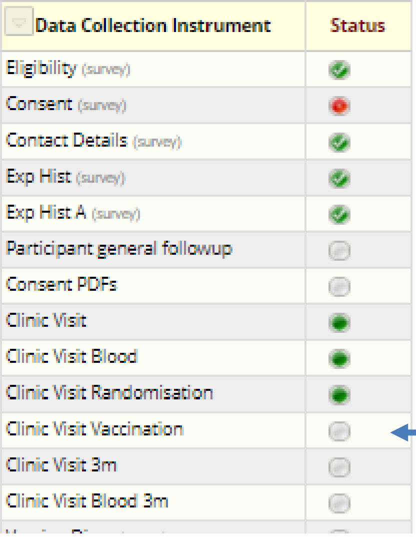

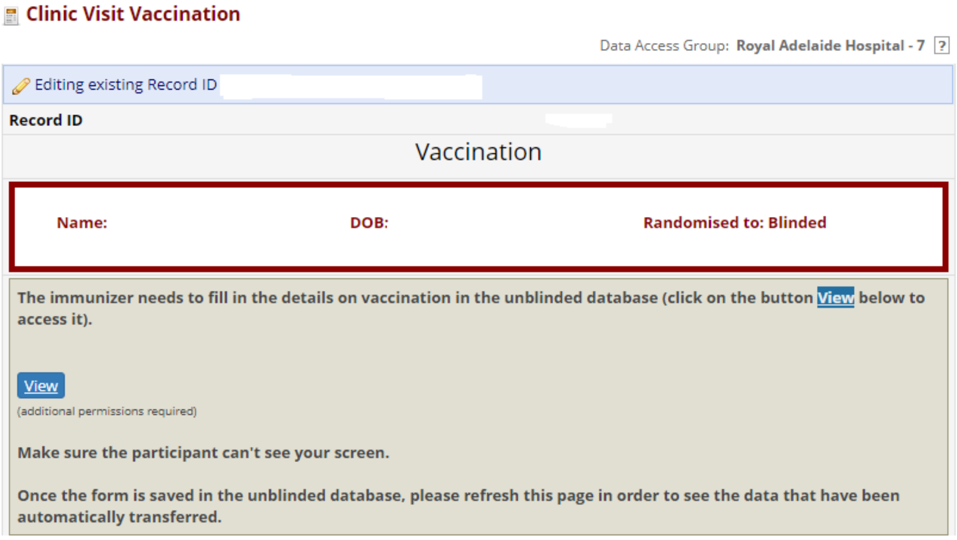

15. Click on the blue 'View' button – this takes the vaccinator to the vaccination page in the Unblinded project ***BRACE UNBLINDED Vaccination*** with the randomisation allocation (BCG or placebo) – Only those with access will be able to see this. Do NOT tell the participant which group they have been allocated to.

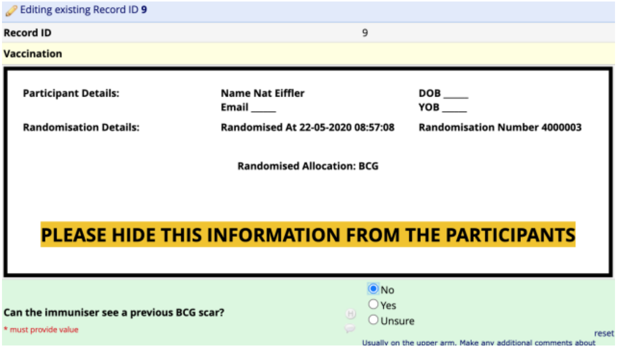

16. Check whether participant has previously received BCG vaccination. If yes, you will need to sight any visible scar (usually upper left shoulder, although some countries

(including Brazil) give BCG in the upper right shoulder – please check both arms), and record against first question. Record anything of note under "**General Comments**".

17. Route and method of administration

a. The time and date of resuspension of the vial, batch identifier, immunisation date/time, any problems with immunisation should be entered in the participants' study record (see below).
b. Both the vaccinator and the participant receiving the vaccine should wear protective eyewear. Eye splashes can ulcerate. If eyes are splashed, wash the eyes with saline or water immediately and contact the sponsor-investigator (or delegate) to decide on management and follow-up.
c. The injection site should be cleaned using a non-alcohol-based antiseptic and allowed to dry. Alcohol antiseptics should not be used prior to administration.
d. If alcohol is used to swab the skin, it must be allowed to evaporate before the vaccine or placebo is injected.
e. The vaccine or placebo must be given strictly intradermally, approximately one third down the upper left arm (unless tattoo or other issue prevents injection in that arm) corresponding to the area of the distal insertion of the deltoid muscle, as follows:
    i. The skin is stretched between thumb and forefinger (see below diagram).
    ii. The needle should be almost parallel with the skin surface and slowly inserted (bevel upwards), approximately 2 mm into the superficial layers of the dermis. The needle should be visible through the epidermis during insertion.
    iii. 0.1ml of the vaccine or placebo should be administered and given slowly.

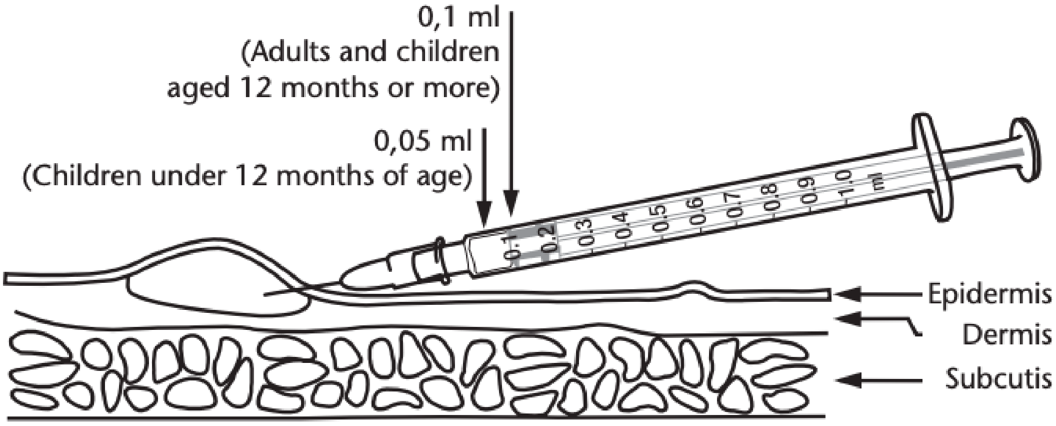

Source: BCG vaccine AJV Package leaflet information for the user (AJVaccines)

    iv. The mixed vaccine should be administered with a syringe of 1 mL graduated into hundredths of millilitre (1/100) fitted with a short bevel syringe needle (25G or 26G preferred, but up to 30G is acceptable).

v. A new syringe and needle are used for each dose. No needle should be left inside the vials of BCG or placebo.

vi. You should feel considerable resistance as you give the injection. If there is no resistance, the needle may be in the subcutaneous tissues.

vii. Please only give one dose intradermally. If the injection is not intradermal, you can withdraw the needle and repeat at a new site. **Please limit to maximum total two attempts.**
Note: if more than two doses are given, it is a protocol deviation and a Protocol Deviation and Violation form needs to be completed in REDCap.
If 2 doses are given, this is not considered a protocol deviation, but this must still be documented in the BRACE UNBLINDED Vaccination form.

viii. A raised, blanched papule/bleb of about 7 mm diameter (looks like orange peel) at the needle point is a sign of correct injection.

ix. The injection site is best left uncovered to facilitate healing.

x. Please note: Jet injectors or multiple puncture devices should not be used to administer the vaccine.

18. Once the vaccine or placebo has been administered (see instructions below) click "Vaccine given?" "Yes". The following questions will then appear.

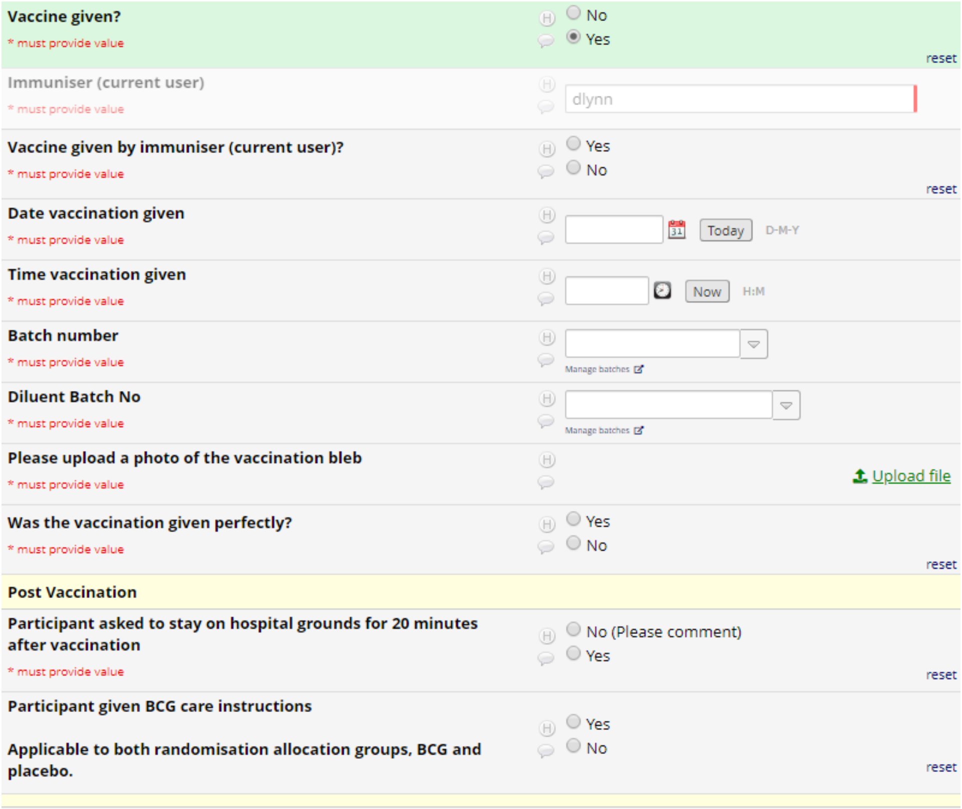

Vaccine given? * must provide value — No / Yes (selected) — reset
Immuniser (current user) * must provide value — dlynn
Vaccine given by immuniser (current user)? * must provide value — Yes / No — reset
Date vaccination given * must provide value — Today D-M-Y
Time vaccination given * must provide value — Now H:M
Batch number * must provide value — Manage batches
Diluent Batch No * must provide value — Manage batches
Please upload a photo of the vaccination bleb * must provide value — Upload file
Was the vaccination given perfectly? * must provide value — Yes / No — reset
Post Vaccination
Participant asked to stay on hospital grounds for 20 minutes after vaccination * must provide value — No (Please comment) / Yes — reset
Participant given BCG care instructions
Applicable to both randomisation allocation groups, BCG and placebo. — Yes / No — reset

19. Answer these questions and record the date and time of vaccination (BCG/placebo), batch number of the vaccine and diluent used.

20. [Include this step if your site requires a hard copy log of drug and diluent dispensing]. Drug and diluent dispensing are also to be logged in the paper logs provided by [site name] on a vial by vial basis. Record left over resuspended vaccine vials >4hrs since resuspension that are disposed of. All drugs must be fully accounted for.

21. After the vaccine/placebo has been administered, complete "**upload a photo of the vaccination bleb**" in REDCap. This must be done, even if placebo has been administered.

    Ask participant to: hold arm parallel to ground and turn head away, place 'Vaccination Site Ruler' (shown below) next to bleb, hold the tablet camera directly above bleb and take photograph of **arm only**.

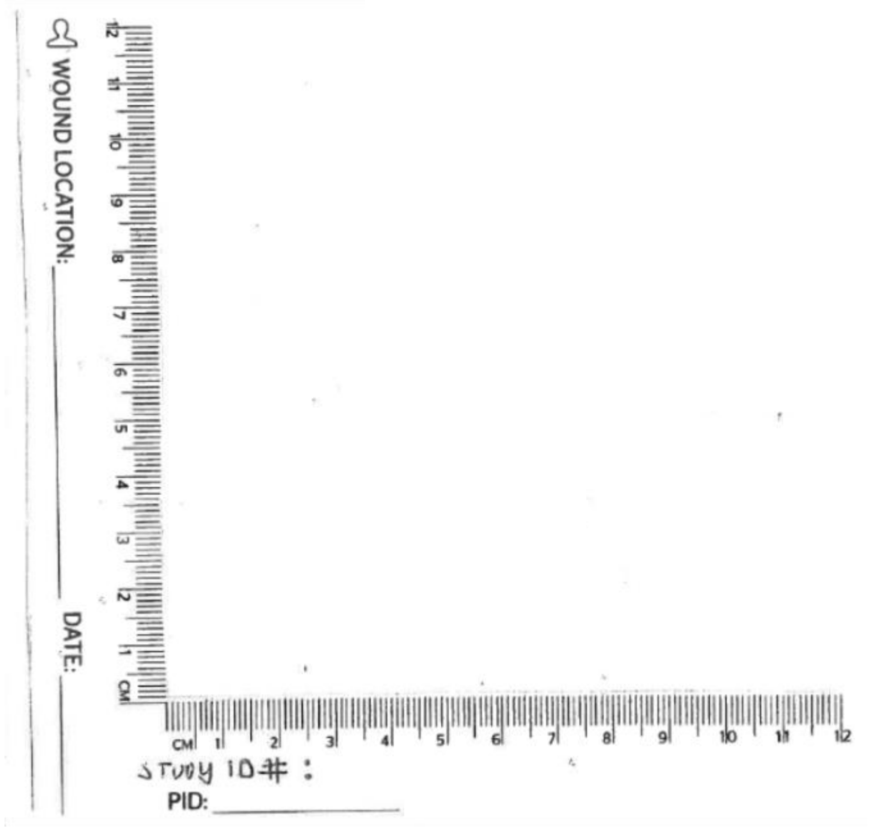

22. Give the participant their own Vaccination Site Ruler to take home. Teach the participant how to use the Ruler accurately and how to take photos of their vaccination site for their Vaccine Diary.

    Ideally, this Vaccination Site Ruler will be printed onto hard paper for the participant so that it retains its form. When printing the ruler, make sure that it is to scale.

23. If the vaccine/placebo was not given perfectly (e.g. no bleb formed, bleb very small or other) tick 'no' in response to 'Was the vaccination given perfectly?' and record the reason why in the tick box and comment section 'Please give details' (see example below).

(Note – a small amount of leakage following vaccination is normal).

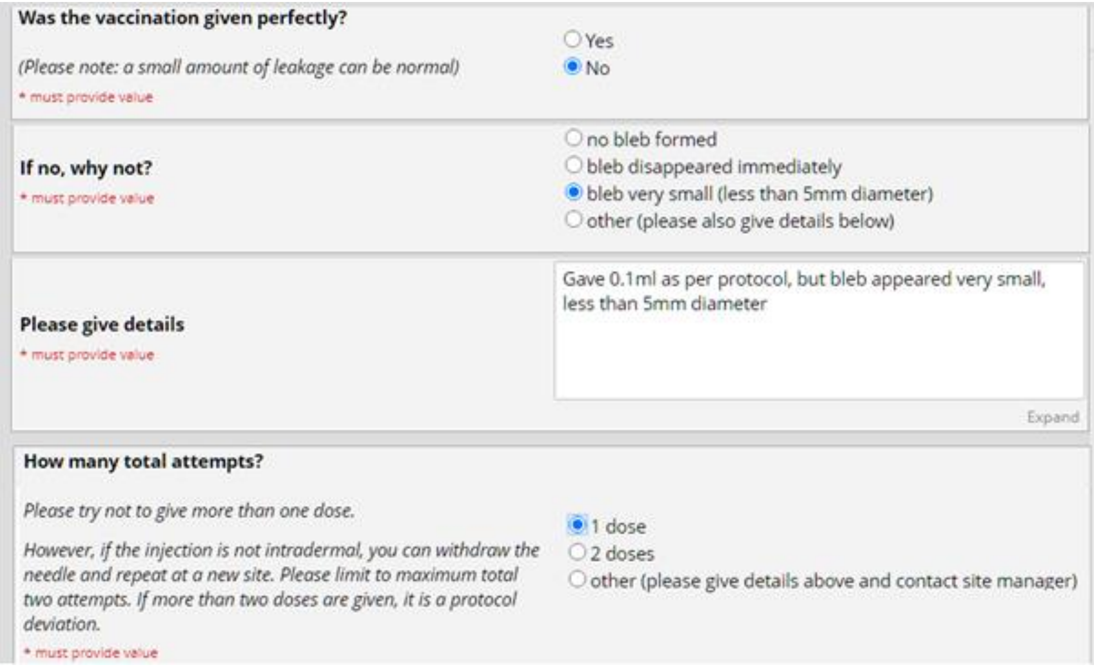

24. Any variations are to be documented, and standard procedures followed regarding the need for re-administration, notification to Sponsor-Investigator (or delegate), and fill out a 'Protocol Deviation And Violation' form on REDCap according to the situation.

25. Select **"Complete, Save & Exit".**

26. Return to the tab in the **BRACE EConsent and Randomisation** project and press 'Refresh'. The fields of vaccination given, previous BCG scar, date and time, will now automatically populate in this project from **BRACE Unblinded vaccination** project. Save this form as complete.

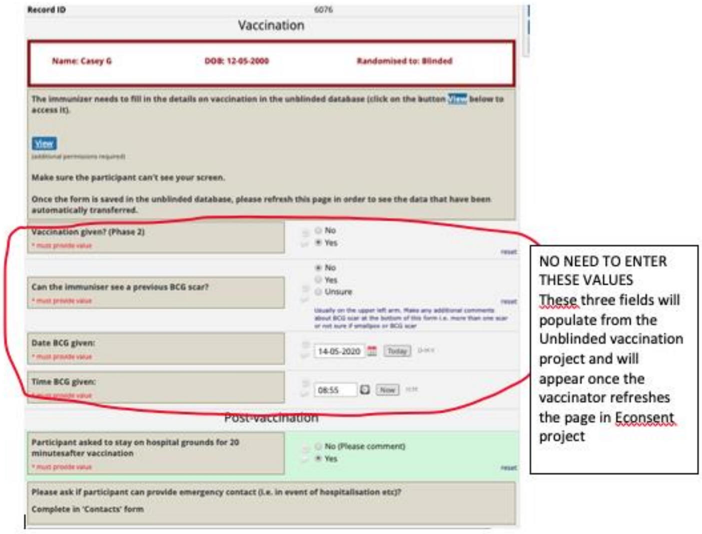

27. [Include this step if your site requires participants to remain at trial clinic for 20 minutes for observation].

    Participants should be given a sticker advising that they have been "***immunised as part of the BRACE trial***" and directed to the observation area and advise that they will need to remain here for 20 minutes.

28. The vaccinator will need to provide the participant with verbal and printed information (in the form of the MCRI "What to Expect" Webpage) regarding what to expect following the BCG vaccine, care of the injection site, when to seek medical advice, who to contact for advice, and how to take best take a picture of the vaccination site for the Vaccine survey: This information can be found: https://www.mcri.edu.au/BRACE/what-to-expect.

29. During this 20-minute period, staff from [site name] will double check that the participant has downloaded the "Trial Symptom Tracker" App and explain what is required from them and the difference between the App and the Vaccine Diary.

Appendix 1: Placebo and Study Drug Vials

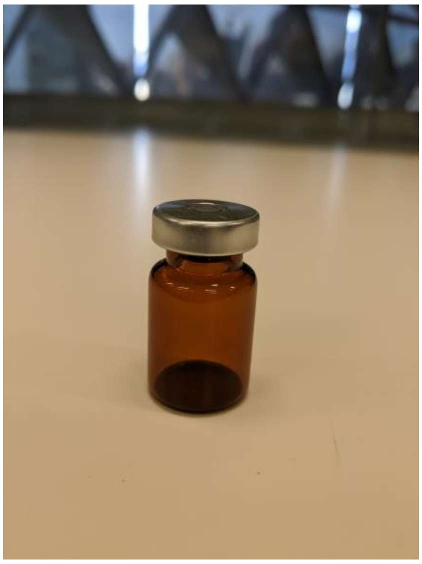

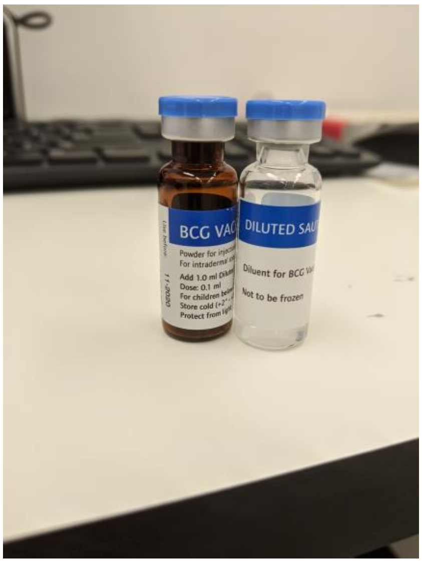

Storage and dispensing of BCG vaccine SSI, and Placebo

BCG vaccine SSI

- Store between 2°C - 8°C AT ALL TIMES
- Store in the original package in order to protect from light
- Do not freeze
- Do not use the vaccine after the expiry date which is stated on the carton as "EXP" and refers to the last day of the month listed
- Any unused vaccine at the end of the study, meaning vaccines unused after the last dosing of the last participant will be disposed of according to local regulations

Placebo - sodium chloride 0.9%

- Store less than 25°C
- Do not use after the expiry date which is stated on the carton and ampoule as "EXP" and refers to the last day of the month listed
- Any unused sodium chloride 0.9% at the end of the study, unused ampoules after the last dosing of the last participant will be disposed of according to local regulations.
