# Supplementary material for: Factors influencing scar formation following Bacille Calmette-Guérin (BCG) vaccination
Source: Heliyon. 2023 Apr 6;9(4):e15241. doi: 10.1016/j.heliyon.2023.e15241 (PMC10126857; doi:10.1016/j.heliyon.2023.e15241)
Supplement: Multimedia component 2 [file mmc2.pdf]

Supplemental Material 1: 12-month vaccine site questionnaire

Vaccine site reaction

We would love to see a picture of your vaccination site, even if it is not visible anymore. How to take the best picture:
- Attach a standard-sized object to your upper arm (e.g. coin or measuring tape or ruler) using rolled up sticky tape or BluTack, adjacent to the vaccination site.
- Hold your phone approx. 15cm away from the area being photographed.
- Ensure the entire injection site and coin are in the photo and in focus.

Please upload your photo (vaccine site) here.

Do you have a scar (or bump or mark) at your BRACE vaccination site?
(Please answer 'no' if there is nothing to see)

○ Yes
○ No

Which of the following best describes the vaccination site today?
(Please do not hesitate to contact us via email or phone if you are concerned.)

○ Skin colour mark without redness (normal scar formation)
○ Red mark
○ Red mark with discharge
○ Red mark with crusting
○ Ulcer (open sore)
○ Vaccination site still looks 'angry' with swelling and/or redness all around it
○ An abnormal thick scar
○ Other

If other, please describe:

The scar (or bump or mark) is:

○ Palpable only, not visible
○ Visible only, not palpable
○ Visible and palpable
○ Visible and palpable with crust

What do you think about your scar?

○ I don't mind having the scar at all
○ I would rather not have a scar, but understand this is unavoidable
○ I'm dissatisfied with the scar

Why are you dissatisfied with your scar?

○ I didn't expect to have a scar
○ It is worse than I expected
○ Other

If other, please describe:

Do you regret having the vaccine because of the scar?

○ No, I don't regret having the vaccine
○ Yes, I regret having the vaccine
