# Supplementary material for: Factors influencing scar formation following Bacille Calmette-Guérin (BCG) vaccination
Source: Heliyon. 2023 Apr 6;9(4):e15241. doi: 10.1016/j.heliyon.2023.e15241 (PMC10126857; doi:10.1016/j.heliyon.2023.e15241)
Supplement: Multimedia component 3 [file mmc3.docx]

**Supplemental Material 3**

| **What do you think about your scar ?** | n =2341 |
| --- | --- |
| *I don't mind having the scar at all* | 1806 (77%) |
| *I would rather not have a scar, but understand this is unavoidable* | 472 (20%) |
| *I'm dissatisfied with the scar* | 59 (3%) |
| *Unknown** | 4 (<1%) |

*4 participants with missing data

| **Why are you dissatisfied with the scar?** | n =59 |
| --- | --- |
| *I didn't expect to have a scar* | 10 (17%) |
| *It is worse than I expected* | 46 (78%) |
| *Other** | 3 (5%) |

*scar location, odd appearance, different appearance (purple/red) to that expected.

| **Do you regret having the vaccine because of the scar?** | Total | Australia | Brazil | Netherlands | Spain | UK |
| --- | --- | --- | --- | --- | --- | --- |
|  | n =2341* | n =1003* | n =1032* | n =187 | n =52 | n =67 |
| *No, I don’t regret having the vaccine* | 2242 (96%) | 931 (93%) | 1023 (99%) | 173 (93%) | 51 (98%) | 64 (96%) |
| *Yes, I regret having the vaccine* | 88 (4%) | 66 (7%) | 4 (<1%) | 14 (7%) | 1 (2%) | 3 (4%) |

*11 participants with missing data (6 in Australia, 5 in Brazil)

# Supplemental Table 1. Sensitivity analysis; factors investigated for association with BCG scar formation (Australia)

Abbreviations: BCG, Bacille Calmette-Guérin; BMI, body mass index; LTBI, latent tuberculosis infection; NA, not applicable; OR, odds ratio; TST, tuberculin skin test

*Wheal response (yes/no) analysed for participants who received one BCG dose only.

Significant factors (p-value < 0.2) resulting from the univariate logistic regression analysis were included as possible covariates in a multivariate logistic regression model. The model presented in the table was created using backward stepwise exclusion of factors with p-value > 0.05, using sequential model testing.

|  | **Total** |  | **Scar prevalence** | |  |
| --- | --- | --- | --- | --- | --- |
| **Factor** | **BCG** |  | Univariate | Multivariate |  |
|  | **n=1380** | n/N (%) | OR (95% CI) | OR (95% CI) |  |
| Sex |  |  |  |  |  |
| Male | 353 | 219 (62.0) | 1 (reference) | 1 (reference) |  |
| Female | 1027 | 784 (76.3) | 1.97 (1.52-2.56), p<0.001 | 2.04 (1.54-2.71), p<0.001 |  |
| Age |  |  |  |  |  |
| 18-49 | 912 | 701 (76.9) | 1 (reference) | 1 (reference) |  |
| ≥50 | 468 | 302 (64.5) | 0.55 (0.43-0.70), p<0.001 | 0.41 (0.31-0.54), p<0.001 |  |
| Nutritional status (BMI) |  |  |  |  |  |
| Normal weight (18.5-24.9) | 621 | 471 (75.8) | 1 (reference) | - |  |
| Underweight (<18.5) | 11 | 7 (63.6) | 0.56 (0.16-1.93), p=0.4 |  |  |
| Pre-obesity (25.0-29.9) | 451 | 311 (69.0) | 0.71 (0.54-0.93), p=0.01 |  |  |
| Obesity class I (30.0-34.9) | 163 | 114 (69.9) | 0.74 (0.51-1.09), p=0.1 |  |  |
| Obesity class II (35.0-39.9) | 62 | 47 (75.8) | 1.00 (0.54-1.84), p=0.9 |  |  |
| Obesity class III (>40) | 32 | 26 (81.3) | 1.38 (0.56-3.42), p=0.5 |  |  |
| Unknown | 40 | 27 (67.5) | NA |  |  |
| Smoker |  |  |  |  |  |
| No | 1298 | 948 (73.0) | 1 (reference) | - |  |
| Yes | 82 | 55 (67.1) | 0.75 (0.47-1.21), p=0.2 |  |  |
| Diabetes |  |  |  |  |  |
| No | 1359 | 988 (72.7) | 1 (reference) | - |  |
| Yes | 21 | 15 (71.4) | 0.94 (0.36-2.44), p=0.9 |  |  |
| Chronic respiratory disease |  |  |  |  |  |
| No | 1276 | 920 (72.1) | 1 (reference) | - |  |
| Yes | 104 | 83 (79.8) | 1.53 (0.93-2.51), p=0.1 |  |  |
| Chronic cardiovascular disease |  |  |  |  |  |
| No | 1274 | 934 (73.3) | 1 (reference) | - |  |
| Yes | 106 | 69 (65.1) | 0.68 (0.45-1.03), p=0.07 |  |  |
| BCG history |  |  |  |  |  |
| 1st BCG | 651 | 463 (71.1) | 1 (reference) | 1 (reference) |  |
| BCG revaccination | 729 | 540 (74.1) | 1.16 (0.92-1.47), p=0.2 | 1.58 (1.19-2.08), p=0.001 |  |
| Previous known LTBI |  |  |  |  |  |
| No | 1353 | 983 (72.7) | 1 (reference) | - |  |
| Yes | 15 | 9 (60.0) | 0.56 (0.20-1.60), p=0.3 |  |  |
| Unknown | 12 | 11 (91.7) | NA |  |  |
| Previous TST |  |  |  |  |  |
| Negative/None | 1021 | 739 (72.4) | 1 (reference) | - |  |
| Positive (>5mm) | 115 | 88 (76.5) | 1.24 (0.79-1.96), p=0.3 |  |  |
| Unknown | 244 | 176 (72.1) | NA |  |  |
| BCG batch |  |  |  |  |  |
| 118006D | 591 | 431 (72.9) | 1.02 (0.80-1.30), p=0.9 | - |  |
| 118017F | 789 | 572 (72.5) | 0.98 (0.77-1.24), p=0.9 |  |  |
| Co-administered influenza vaccine† |  |  |  |  |  |
| No | 192 | 152 (79.2) | 1 (reference) | 1 (reference) |  |
| Yes | 1188 | 851 (71.6) | 0.66 (0.46-0.96), p=0.03 | 0.57 (0.39-0.84), p<0.01 |  |
| Post-injection wheal* |  |  |  |  |  |
| Yes | 1231 | 899 (73.0) | 1 (reference) | 1 (reference) |  |
| No | 19 | 11 (57.9) | 0.51 (0.20-1.27), p=0.1 | 0.34 (0.13-0.90), p=0.03 |  |
| Unknown | 130 | 93 (71.5) | NA | - |  |
| Vaccinator experience |  |  |  |  | |
| ≥20 vaccinees | 1199 | 864 (72.1) | 1 (reference) | - | |
| 0-19 vaccinees | 181 | 139 (76.8) | 1.28 (0.89-1.85), p=0.2 |  | |

# Supplemental Table 2. Sensitivity analysis; factors investigated for association with BCG scar formation (Brazil)

Abbreviations: BCG, Bacille Calmette-Guérin; BMI, body mass index; LTBI, latent tuberculosis infection; NA, not applicable; OR, odds ratio; TST, tuberculin skin test

*Wheal response (yes/no) analysed for participants who received one BCG dose only.

†Variable omitted from the logistic regression model due to perfect prediction of a scar outcome.

Significant factors (p-value < 0.2) resulting from the univariate logistic regression analysis were included as possible covariates in a multivariate logistic regression model. The model presented in the table was created using backward stepwise exclusion of factors with p-value > 0.05, using sequential model testing.

|  | **Total** |  | **Scar prevalence** | |  |
| --- | --- | --- | --- | --- | --- |
| **Factor** | **BCG** |  | Univariate | Multivariate |  |
|  | **n=1222** | n/N (%) | OR (95% CI) | OR (95% CI) |  |
| Sex |  |  |  |  |  |
| Male | 350 | 279 (79.7) | 1 (reference) | 1 (reference) |  |
| Female | 872 | 753 (86.3) | 1.61 (1.16-2.23), p<0.01 | 1.65 (1.18-2.30), p<0.01 |  |
| Age |  |  |  |  |  |
| 18-49 | 977 | 856 (87.6) | 1 (reference) | 1 (reference) |  |
| ≥50 | 245 | 176 (71.8) | 0.36 (0.26-0.51), p<0.001 | 0.37 (0.26-0.52), p<0.001 |  |
| Nutritional status (BMI) |  |  |  |  |  |
| Normal weight (18.5-24.9) | 387 | 327 (84.4) | 1 (reference) | - |  |
| Underweight (<18.5) | 18 | 15 (83.3) | 0.59 (0.26-3.27), p=0.9 |  |  |
| Pre-obesity (25.0-29.9) | 498 | 418 (83.9) | 0.96 (0.67-1.38), p=0.8 |  |  |
| Obesity class I (30.0-34.9) | 212 | 187 (88.2) | 1.37 (0.83-2.26), p=0.2 |  |  |
| Obesity class II (35.0-39.9) | 81 | 66 (81.5) | 0.81 (0.43-1.51), p=0.5 |  |  |
| Obesity class III (>40) | 21 | 14 (66.7) | 0.37 (0.14-0.95), p=0.04 |  |  |
| Unknown | 5 | 5 (100.0) | NA |  |  |
| Smoker |  |  |  |  |  |
| No | 1090 | 916 (84.0) | 1 (reference) | - |  |
| Yes | 132 | 116 (87.9) | 1.38 (0.80-2.38), p=0.3 |  |  |
| Diabetes |  |  |  |  |  |
| No | 1167 | 988 (84.7) | 1 (reference) | - |  |
| Yes | 55 | 44 (80.0) | 0.72 (0.37-1.43), p=0.4 |  |  |
| Chronic respiratory disease |  |  |  |  |  |
| No | 1162 | 980 (84.3) | 1 (reference) | - |  |
| Yes | 60 | 52 (86.7) | 1.21 (0.56-2.58), p=0.6 |  |  |
| Chronic cardiovascular disease |  |  |  |  |  |
| No | 1047 | 894 (85.4) | 1 (reference) | - |  |
| Yes | 175 | 138 (78.9) | 0.64 (0.43-0.95), p=0.03 |  |  |
| BCG history |  |  |  |  |  |
| 1st BCG | 40 | 31 (77.5) | 1 (reference) | ~~-~~ |  |
| BCG revaccination | 1182 | 1001 (84.7) | 1.61 (0.75-3.43), p=0.2 |  |  |
| Previous known LTBI |  |  |  |  |  |
| No | 1218 | 1028 (84.4) | 1 (reference) | - |  |
| Yes | 1 | 1 (100.0) | NA† |  |  |
| Unknown | 3 | 3 (100.0) | NA |  |  |
| Previous TST |  |  |  |  |  |
| Negative/None | 1150 | 970 (84.3) | 1 (reference) | - |  |
| Positive (>5mm) | 42 | 38 (90.5) | 1.76 (0.62-5.00), p=0.3 |  |  |
| Unknown | 30 | 24 (80.0) | NA |  |  |
| BCG batch |  |  |  |  |  |
| 118019D | 658 | 536 (81.5) | 0.60 (0.44-0.83), p<0.01 | 0.63 (0.45-0.87), p<0.01 |  |
| 119039B | 3 | 3 (100.0) | NA† | NA† |  |
| 119053A | 557 | 491 (88.2) | 1.71 (1.23-2.35), p=0.001 | 1.58 (1.13-2.19), p<0.01 |  |
| Unknown | 4 | 2 (50.0) | NA | NA |  |
| Post-injection wheal* |  |  |  |  |  |
| Yes | 1212 | 1024 (84.4) | 1 (reference) |  |  |
| No | 6 | 6 (100.0) | NA† |  |  |
| Unknown | 4 | 2 (50.0) | NA |  |  |
| Vaccinator experience |  |  |  |  | |
| ≥20 vaccinees | 1098 | 926 (84.3) | 1 (reference) | - | |
| 0-19 vaccinees | 124 | 106 (85.5) | 1.09 (0.65-1.85), p=0.7 |  | |
